# Medical Diseases of Infancy and Childhood

**Published:** 1900-12

**Authors:** 


MEDICAL DISEASES OF INFANCY AND CHILDHOOD. By DAWSON WILLIAMS, M D., Physician to the East London Hospital for Children. New (2d) edition. Specially revised for America by F. S Churchill, A. B., M. D., Instructor in Diseases of Children, Rush Medical College. Octavo, pp. 538, 52 illustrations and 2 colored plates Philadelphia and New York: Lea Brothers & Co. 1900. [Cloth, $3.50 net.]

When the first edition of this book appeared two years ago it was not one likely to appeal greatly to the American student of pediatrics, for, in this branch of medicine at least, we do things better here than in Great Britain. The second edition has, however, undergone a thorough revision at the hands of the American editor with the result of making it an interesting, valuable and up-to-date contribution to the works on diseases of children.

Extensive additions have been made throughout the work. The chapter on infant feeding, which as it appeared in the older edition, was of but little value, has been thoroughly modernised and Americanised. All through the volume the careful hand of the editor in supplementing the work of the author is evident, and this without any abbreviation or alteration of the original text. The editor's contributions are of great value, too, and in many places necessary to make the book what it now is, a safe guide in pediatric practice. Many illustrations have been added, among them two colored plates, one of the latter, showing the varieties of blood corpuscles, being taken from Park's surgery.

The style of the English author is clear, his description is accurate, his experience wide. The subject of treatment is well handled, being rational and modern. The prescriptions given have been confined to the U. S. Pharmacopeia.

The publishers present a far more attractive volume than the old one, the duodecimo being changed to an octavo, and the type and paper both made pleasanter to the eye.—M. J. F.

